# Terpene synthases in cucumber (*Cucumis sativus*) and their contribution to herbivore‐induced volatile terpenoid emission

**DOI:** 10.1111/nph.17814

**Published:** 2021-11-10

**Authors:** Jun He, Francel Verstappen, Ao Jiao, Marcel Dicke, Harro J. Bouwmeester, Iris F. Kappers

**Affiliations:** ^1^ Laboratory of Plant Physiology Plant Sciences Group Wageningen University & Research 6700AA Wageningen the Netherlands; ^2^ Citrus Research Institute Southwest University 400712 Chongqing China; ^3^ Laboratory of Entomology Plant Sciences Group Wageningen University & Research 6700AA Wageningen the Netherlands; ^4^ Plant Hormone Biology Group Swammerdam Institute for Life Sciences University of Amsterdam 1000BE Amsterdam the Netherlands

**Keywords:** Aphids, circadian rhythm, cucumber (*Cucumis sativus*), herbivore‐induced terpenoids, spider mites, terpene synthases, thrips

## Abstract

- Terpenoids play important roles in flavour, pollinator attraction and defence of plants. In cucumber (*Cucumis sativus*) they are important components of the herbivore‐induced plant volatile blend that attracts natural enemies of herbivores.
- We annotated the cucumber *TERPENE SYNTHASE* gene (*CsTPS*) family and characterized their involvement in the response towards herbivores with different feeding guilds using a combined molecular and biochemical approach.
- Transcripts of multiple *CsTPS* genes were upregulated in leaves upon herbivory and the products generated by the expressed proteins match the terpenoids recorded in the volatile blend released by herbivore‐damaged leaves. Spatial and temporal analysis of the promoter activity of *CsTPS* genes showed that cell content‐feeding spider mites (*Tetranychus urticae*) and thrips (*Frankliniella occidentalis*) induced promoter activity of *CsTPS9* and *CsTPS19* within hours after initiation of infestation, while phloem‐feeding aphids (*Myzus persicae*) induced *CsTPS2* promoter activity.
- Our findings offer detailed insights into the involvement of the *TPS* gene family in the dynamics and fine‐tuning of the emission of herbivore‐induced plant volatiles in cucumber, and open a new avenue to understand molecular mechanisms that affect plant–herbivore interactions.

## Introduction

Specialized metabolites modulate interactions of plants with their biotic environment. Numerous endogenous compounds function in direct defence as toxins and repellents towards herbivores and pathogens (Schoonhoven *et al*., 2005; Hopkins *et al*., 2009). Volatile compounds have additional functions as attractants for pollinators and carnivorous enemies of herbivores, as well as in inter‐ and intra‐plant communication (Pichersky & Gershenzon, 2002; Degenhardt *et al*., 2003; Kappers *et al*., 2005; Dudareva & Pichersky, 2020). Upon herbivory, the plant’s specialized metabolome changes depending on the feeding habit of the infesting herbivore. For example, chewing caterpillars inflict significant damage, while aphids cause only little tissue damage, manoeuvring their flexible stylet intercellularly through the epidermis and mesophyll to reach the phloem (Kloth *et al*., 2016). Other herbivores inflict moderate damage, including spider mite and thrips that pierce mesophyll plant cells and feed on their contents. In addition to the mechanical wounding inflicted, cues in the herbivore’s oral secretion trigger a cascade of reactions including early Ca^2+^ signalling and a burst of reactive oxygen species (Maffei *et al*., 2007), followed by changes in the concentrations of phytohormones (Wu & Baldwin, 2010). The synthesis, perception and crosstalk of these hormones, the transcription factors involved and their target genes together constitute a complicated signal‐transduction network through which the plant metabolome and therefore the defensive state of the plant is rearranged.

Terpenoids represent the most diverse group of plant specialized metabolites (Aharoni *et al*., 2005) and many have roles in the interaction between plants and their environment. Terpenoids are the main constituents of the blend of leaf‐emitted volatiles after oviposition, herbivory and wounding that induce endogenous jasmonic acid (JA) (Bohlmann *et al*., 2000; Herde *et al*., 2008; Cao *et al*., 2010; Hilker & Fatouros, 2015), and nonvolatile terpenoids increase in plant organs upon exposure to (a)biotic stresses (Bohlmann *et al*., 2000; Balkema‐Boomstra *et al*., 2003; Nagegowda, 2010).

Terpenoids are composed of isoprenoid units originating from either the mevalonate (MVA) or the 2‐C‐methylerythritol‐4‐phosphate (MEP) pathway. The genes encoding terpene synthases (TPSs) are structurally related and constitute a medium‐sized gene family occurring across the plant kingdom. For example, the Arabidopsis genome contains 32 genes encoding functional TPSs (Chen *et al*., 2011). By contrast, the *Vitis vinifera* genome contains 152 *TPS* genes (Martin *et al*., 2010), while the moss *Physcomitrella patens* contains only a single one (Hofberger *et al*., 2015). Furthermore, transcription of *TPS* genes was reported to be upregulated by herbivory in various species, including Arabidopsis (de Vos *et al*., 2005; Zhurov *et al*., 2014), tomato (Kant *et al*., 2004; Martel *et al*., 2015), maize (Schnee *et al*., 2002) and legumes (Arimura *et al*., 2004).

Plant defences against biotic stressors can be affected by internal and external factors including light and the circadian clock. In Arabidopsis, the expression of more than 40% of the genes induced by mechanical damage peaks at dusk and over 80% of the genes is suppressed at dawn (Walley *et al*., 2007). Arabidopsis plants grown under a similar light : dark rhythm as the cabbage looper *Trichoplusia ni*, which has rhythmic feeding behaviour, had increased resistance against this herbivore, while plants grown under an opposite light : dark rhythm as the insect were more susceptible (Goodspeed *et al*., 2012). Both the circadian clock and jasmonates were shown to be essential in maintaining this rhythmic defence.

Cucumber (*Cucumis sativus*) plants infested with *Tetranychus urticae*, two‐spotted spider mites (TSSM), emit a terpenoid‐enriched volatile blend (Takabayashi *et al*., 1994; Mercke *et al*., 2004; Kappers *et al*., 2010) of which (*E*)‐β‐ocimene and (*E,E*)‐4,8,12‐trimethyl‐1,3,7,11‐tridecatetraene (TMTT) were shown to be essential for the attraction of *Phytoseiulus persimilis*, predators of TSSM (Dicke & Sabelis, 1988; Dicke *et al*., 1990, 1990,1990, 1990; Kappers *et al*., 2010, 2011). The cucumber *TPS* gene family has been described and partially characterized, although not in relation to herbivory (Wei *et al*., 2016). An earlier study demonstrated the induction of expression of the cucumber *(E)‐β‐OCIMENE/(E,E)‐α‐FARNESENE SYNTHASE* by TSSM feeding (Mercke *et al*., 2004).

Two‐spotted spider mites predominantly induce JA‐related defences in Arabidopsis (Zhurov *et al*., 2014), tomato (Martel *et al*., 2015) and cucumber (He *et al*., 2020), although young nymphs induce salicylic acid (SA) in tomato (Liu *et al*., 2020) and TSSM induce SA in frijole (He *et al*., 2007), lima bean (Ozawa *et al*., 2000) and pepper (Zhang *et al*., 2020). Western flower thrips (*Frankliniella occidentalis*) is another important generalist pest in many glasshouse crops, inducing JA‐related defences (Shipp *et al*., 2000; Steenbergen *et al*., 2018; Sarde *et al*., 2019). By contrast, the generalist green peach aphid (*Myzus persicae*) is a SA inducer, and the amounts of volatiles emitted by plants in response to phloem feeders such as aphids are generally low (Staudt *et al*., 2010) and sometimes even suppressed upon aphid herbivory (Pineda *et al*., 2013).

Here, we investigated the abundance and composition of the volatile blend of cucumber plants upon feeding by different types of herbivores and characterized the genes encoding the TPSs and their transcriptional regulation responsible for the specificity of the response to herbivores with different feeding guilds.

## Materials and Methods

### Plants and arthropods

*Cucumis sativus* plants (genotype ‘Corona’) were grown in potting soil in a glasshouse (16 h 22°C : 8 h 18 ± 2°C, light : dark) for 3 wk until five true leaves had developed. *Arabidopsis thaliana* Col‐0 (N1092) and *p35S::LUC* in Col‐0 background (N9966) seeds were obtained from the Nottingham Arabidopsis Stock Centre (NASC) and grown in a climate chamber (12 h : 12 h, light : dark, 150 µmol m^−2^ s^−1^, 22°C) for 4 wk. Female adult spider mites (*T. urticae*) were selected from a mass‐rearing on lima beans. Aphids (*Myzus persicae*) were reared on radishes and wingless adults were used for experiments. Thrips (*Frankliniella occidentalis*) were reared on pods of broad bean and 5‐d‐old larvae were used for experiments.

### Assessment of leaf damage

Herbivory damage was ass after 3 d. For mite damage, visual observation of chlorotic spots was supported by trypan blue staining (Keogh *et al*., 1980). Quantification of TSSM and thrips‐induced damage was performed using ImageJ software (imagej.nih.gov/ij) as described by Visschers *et al*. (2018).

### Identification of *CsTPS* genes

The cucumber genome (v.2 assembly; www.icugi.org) was screened for genes related to the terpenoid biosynthetic module using InterProScan (www.ebi.ac.uk/interpro/) according to the method described by Hofberger *et al*. (2015). Genomic regions containing candidate genes and their flanking 4 kb sequence were extracted, re‐annotated and confirmed by Fgenesh (www.softberry.com) and Genewise (www.ebi.ac.uk/) according to the structure of previously reported TPS proteins (Chen *et al*., 2011). The TargetP 1.1 server (Emanuelsson *et al*., 1999) was used for signal peptide prediction, and amino acid alignment of full‐length CsTPS enzymes was constructed using ClustalW (www.genome.jp/tools‐bin/clustalw) and Muscle (www.ebi.ac.uk/Tools/msa/muscle/). A phylogenetic tree was constructed using the maximum‐likelihood method in Mega5 (Tamura *et al*., 2011). Using the previously obtained RNA‐Seq dataset comparing two genotypes that differ in TSSM susceptibility (He *et al*., 2020), reads of genes assigned to the terpenoid biosynthetic module were mapped to assembled sequences to calculate read counts for each unigene. Differentially expressed genes (DEGs) between different experimental conditions were filtered using a Benjamini–Hochberg false discovery rate of 0.05 and a threshold of log_2_‐transformed fold‐changes (treatment/control) > |1.5|.

### Putative *cis*‐element analysis

The 2000 bp intergenic sequences upstream from the initiation start of *CsTPS2*, *CsTPS9* and *CsTPS19* were analysed for the presence of *cis*‐acting elements using the PlantCARE database (http://bioinformatics.psb.ugent.be/webtools/plantcare/). Aligned motifs for each promoter were listed as their distances to the start codon of the gene.

### Volatile collection and analysis

The second fully expanded leaves of 3‐wk‐old cucumber plants were infested with 50 adult TSSM, 10 thrips or 10 aphids, or were left uninfested. Volatile emissions of herbivore‐infested and nontreated plants were collected on Tenax absorbent using dynamic headspace sampling as described by Zhang *et al*. (2020). For semiquantification of volatiles, 1 µl of carvone in 10 μl MeOH was added to each Tenax liner before analysis and the areas under the curve (AUC) were normalized to that of the internal standard. For each experimental condition, volatile emissions were collected from five independent plants.

### RNA isolation and gene expression analysis

Total RNA from cucumber leaves was extracted and reverse‐transcribed for quantitative reverse transcription polymerase chain reaction (qRT‐PCR) analysis as described previously (He *et al*., 2020). Expression levels were normalized to cucumber *β‐Actin* (*Csa6M484600*) and *α‐Tubulin* (*Csa4G000580*) using the ΔΔ*C*_t_ method (Livak & Schmittgen, 2001). Every measurement was performed with five biological replicates. Primer sequences are listed in Supporting Information Table S1.

### Generation and expression of recombinant CsTPSs

Full‐length cDNA sequences of *CsTPS* genes were cloned into the expression vector pACYCDuet (Novagen, Birmingham, UK) and transformed into the *Escherichia coli* strain BL21 (DE3). Primers used to obtain open reading frames are listed in Table S1. Production of heterologous protein was induced using 10 µM farnesyl pyrophosphate (FPP) or geranyl pyrophosphate (GPP) in a 1 ml vial as described previously (Mercke *et al*., 2004). As a negative control, raw protein extracts from *E. coli* expressing the empty pACYCDuet vector with substrates (FPP, GPP or GGPP) were incubated as described earlier. To collect terpenoid products, a 10 mm polydimethylsiloxane (PDMS, film thickness 1 mm) stir bar (Gerstel, Mülheim, Germany) was enclosed in each assay vial for 60 min incubation at 30°C with 250 rpm shaking. Subsequently, the stir bar was briefly rinsed in water, dried under a stream of nitrogen and enclosed in a glass liner for GC‐MS analysis. The PDMS stir bars in between the measurements were cleaned by heating them to 310°C for 40 min with a helium flow. The tentative identification of enzyme‐derived compounds was based on the comparison of mass spectra with those in the NIST 2005, Adams (2007) and Wageningen Mass Spectral Database of Natural Products, as well as experimentally obtained linear retention indices (LRIs). Essential oil of basil (*Ocimum basilicum*) was used to characterize cadinol. For each TPS enzyme and substrate combination, assays were repeated at least twice (*n* = 3) but most often three times (*n* = 4). For all replicates, the major products were similar and in the same order of relative magnitude. Terpenoids were semiquantified by calculating the area under the curve (AUC). To determine efficient mono‐ (sesqui‐) TPS activity, the ratio of the sum of the AUC of all mono‐ (sesqui‐) terpene products to that of all mono‐ (sesqui‐) terpenes, including the nonspecific geraniol (farnesols), was set to be > 50%.

### Construction of cucumber promoter::reporter constructs in Arabidopsis

The 1000 bp intergenic regions upstream of *CsTPS2* (Csa1G066560), *CsTPS9* (Csa2M299880) and *CsTPS19* (Csa3M095040) start codon were PCR‐amplified using Q5 High‐Fidelity DNA polymerase (New England Biolabs, Ipswich, MA, USA) and cloned into vector pMK‐RQ (Thermo Fisher, Waltham, MA, USA). *Agrobacterium tumefaciens* (Agl0) harbouring the promoter::GUC/LUC3300 (Koo *et al*., 2007) fusion reporter constructs were transformed into Arabidopsis Col‐0 plants by floral dipping (Logemann *et al*., 2006). Three independent homozygous T3 transgenic plants were selected for each reporter construct.

### Induction and quantification of ffLUC activity

Four‐week‐old Arabidopsis reporter plants were screened for temporal dynamic imaging of bioluminescence under a diurnal light regime with light ramping to mimic natural light conditions. Plants were acclimatized 24 h before imaging started. The first day of imaging was always under noninduced conditions. Plants were sprayed with 1 mM d‐luciferin (Promega) twice a day. Thirty‐minute interval imaging of firefly‐LUCIFERASE (ffLUC) activity and the determination of relative luminescence profiles were done as described by Van Hoogdalem (2020). Photon emission was depicted with false colour scales, with blue indicating low activity and red indicating high activity.

Transgenic Arabidopsis plants were monitored for reporter activity after various (a)biotic stresses, including mechanical damage (leaf puncturing using a 0.2‐mm‐diameter needle), JA, SA or abscisic acid (ABA) (all 5 μl 1 mM + 0.01% Tween‐20), or individual TSSM, thrips or aphids. Leaves were visually checked for whether herbivores stayed on the leaf where they were introduced. After luminescence measurements were finished, plants were visually checked as to whether herbivores were alive. Experiments were performed with three independent lines per construct and five plants per experimental condition (*n* = 15). Arabidopsis p*35S::ffLUC* reporter plants were used as controls.

## Results

### Cell‐content feeding TSSM and thrips, and phloem‐feeding aphids induce different terpenoid‐enriched volatile profiles

**Fig. 1:**
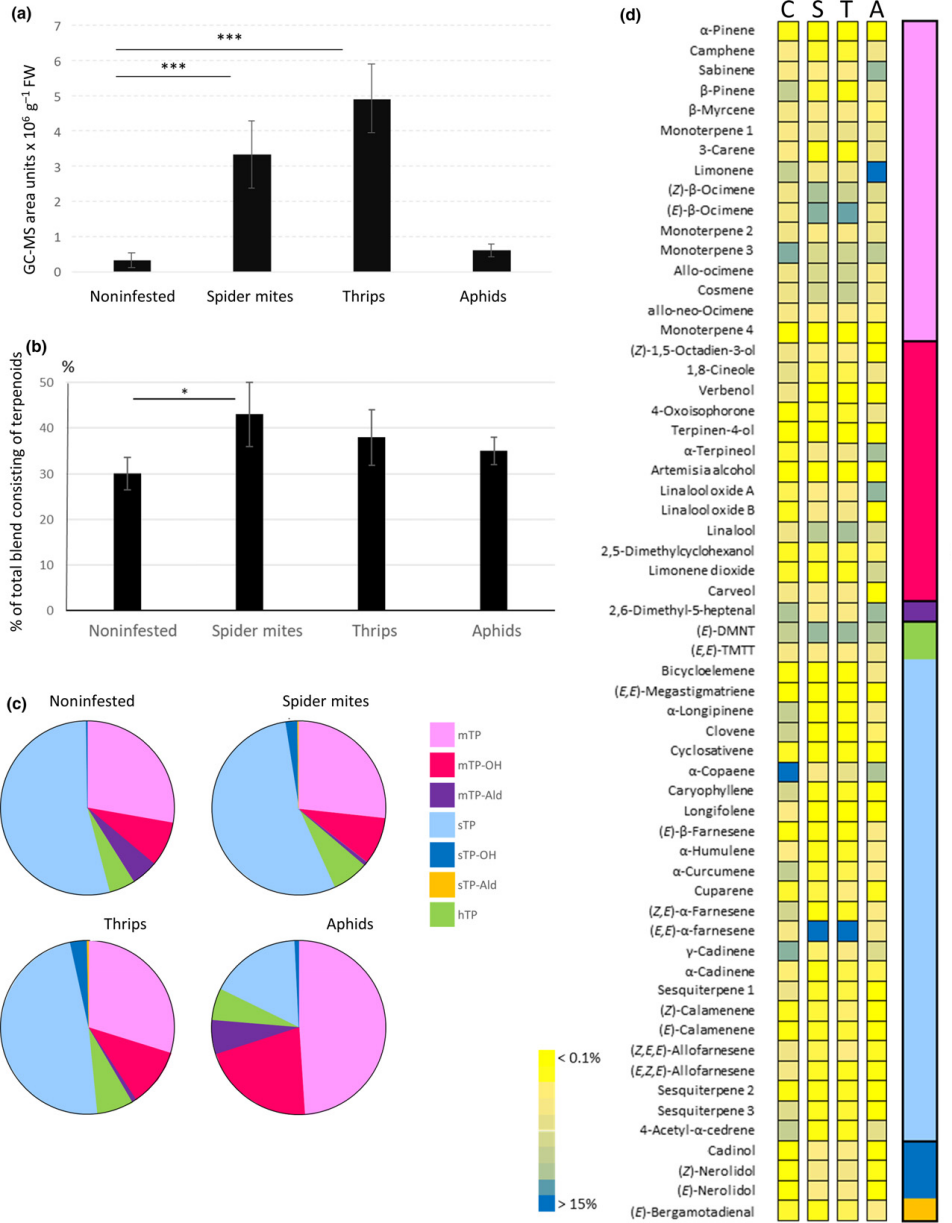
Herbivore‐induced volatile emission in *Cucumis sativus*. (a) Total volatile organic compound (VOC) emission by cucumber plants that were infested for 3 d with *Tetranychus urticae* spider mites (S), *Franklinella occidentalis* thrips (T) or *Myzus persicae* aphids (A) or were left uninfested (C); (b)proportion of terpenoids after 3 d of infestation in the total VOC blend, based on GC‐MS areas under the curve, normalized to internal standard; (c) distribution of monoterpenes (mTP), monoterpene alcohols (mTP‐OH), monoterpene aldehydes (mTP‐Ald); sesquiterpenes (sTP), sesquiterpene alcohols (sTP‐OH), sesquiterpene aldehyde (sTP‐Ald) and homoterpenes (hTP); (d) percentage (% of total GC‐MS signal) of individual compounds to the terpenoid blend. Data represent the means ± SD of five plants. Significance was tested using Mann–Witney *U*‐test (*, *P* < 0.05; ***, *P* < 0.001). (*E*)‐DMNT, (*E*)‐4,8‐dimethylnona‐1,3,7‐triene; (*E,E*)‐TMTT, (*E,E*)‐4,8,12‐trimethyltrideca‐1,3,7,11‐tetraene.

After 3 d of TSSM feeding, damage as chlorotic spots was clearly visible by eye and total volatile emission increased 11‐fold and 16‐fold upon thrips feeding compared with the emission of noninfested plants (Fig. 1a). By contrast, after aphid feeding volatile emission increased less than two‐fold. The volatile blend consisted of green leaf volatiles, benzoates, oximes and terpenoids, of which the latter comprised 30% of the total blend released by noninfested plants (Fig. 1b; Table S2). More than half of all terpenoids emitted by noninfested plants were sesquiterpenoids, of which α‐copaene was the most dominant. The presence and abundance of terpenoids changed depending on the herbivore. The contribution of terpenoids increased to 38% and 43% after 3 d of thrips and TSSM infestation, respectively (Fig. 1b). In both cases, (*E,E*)‐α‐farnesene was the dominant terpenoid followed by (*E*)‐β‐ocimene, linalool, myrcene and (*E*)‐4,8‐dimethylnona‐1,3,7‐triene (DMNT). Interestingly, infestation by both cell‐content feeders increased the contribution of terpene alcohols and aldehydes when compared to the blend of noninfested plants. While aphid infestation resulted in an increased contribution of terpenoids to the total volatile blend, the composition of the induced terpenoid blend differed from that of cell‐content feeders with predominantly monoterpenes and monoterpene alcohols but a lower proportion of sesquiterpenes compared with the blend of noninfested plants (Fig. 1c). Three days after the onset of feeding, limonene was the dominant terpenoid in aphid‐infested plants (Fig. 1d).

### Identification of the terpene biosynthetic module

**Fig. 2:**
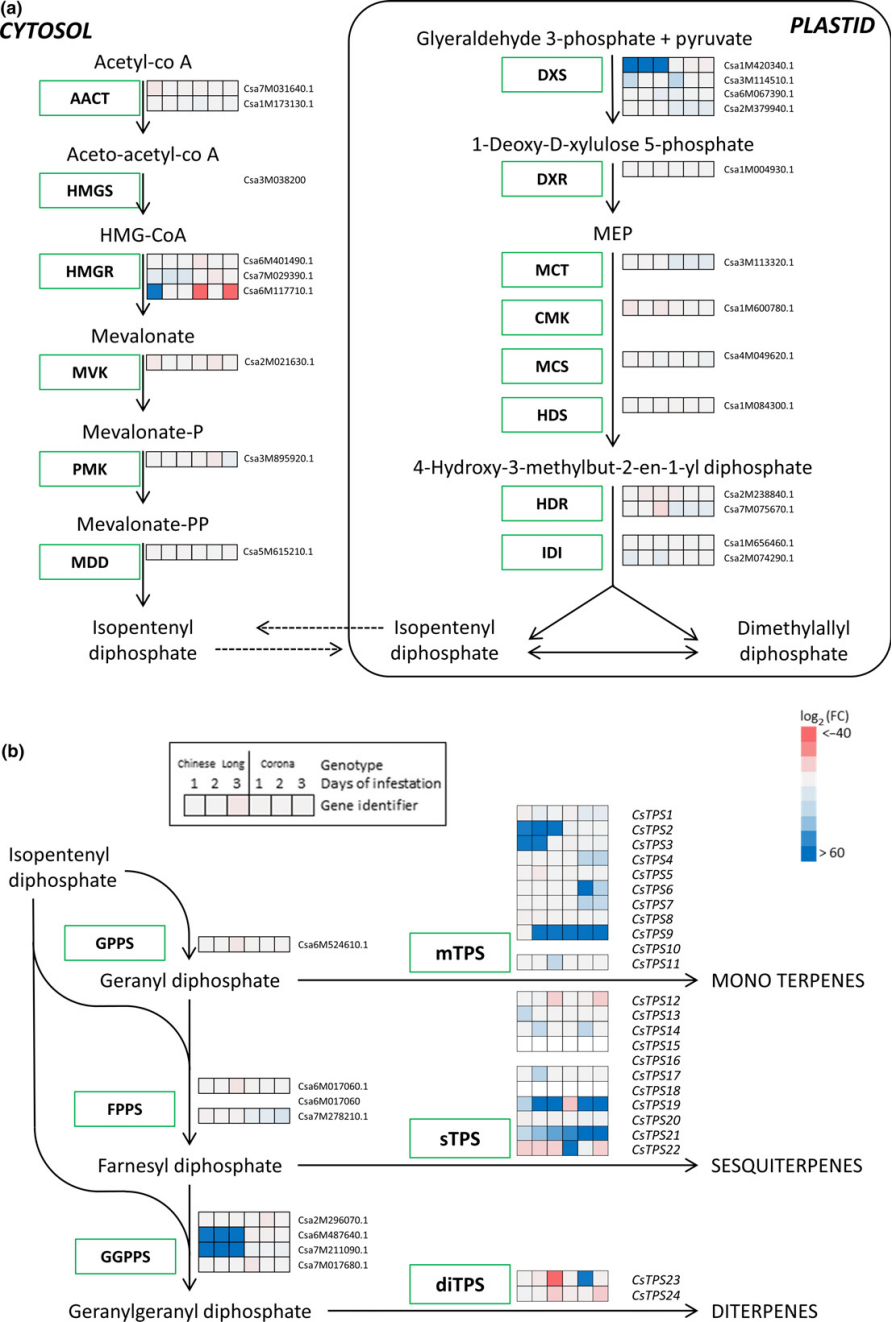
Heat map of the differentially expressed terpenoid biosynthetic module genes in *Cucumis sativus* leaves infested with spider mites. (a) Genes encoding proteins related to 2‐C‐methylerythritol‐4‐phosphate (MEP), mevalonate (MVA) and isoprenoid submodules. (b) Genes encoding proteins related to prenyl transferase and terpene synthase submodules. Values are the log_2_‐fold changes compared with the average expression in noninfested leaves and represent (from left to right): genotype ‘Chinese Long’, infested for 1, 2 and 3 d; genotype ‘Corona’, infested for 1, 2 and 3 d. Pink indicates downregulation of gene expression levels and a strong downregulation is indicated in dark red. Light blue indicates upregulation and dark blue indicates strong upregulation of gene expression. Light grey indicates no changes in gene expression relative to noninfested control. RKPM values are presented in Supporting Information Table S5.

To study the regulation of terpene biosynthesis by herbivory, the cucumber genome was analysed for putative gene models associated with terpene biosynthesis (Fig. 2). Seventy genes could be assigned to one of the six functional modules in terpenoid biosynthesis, including eight prenyl‐transferases, two IPP isomerases and 10 MEP and MVA pathway‐associated genes (Table S3). Additionally, 12 triterpene synthases were detected, and 34 gene models were identified as putative *TPS* genes. From these, 24 full‐length gene models encoding putative proteins with 313 to 813 amino acids and at least five exons were renamed as *CsTPS1‐24* according to their chromosomal position, while three other ones that are too short to encode a functional TPS protein were renamed as *CsTPS25‐27* (Table S4). The majority of *CsTPS* genes were found in clusters located on chromosomes I, II and III, suggesting multiple duplication and neofunctionalization events on these chromosomes (Fig. S1). Phylogenetic analysis classified most cucumber *TPSs* as *TPS*‐a (11 members), *TPS*‐b (eight members) and *TPS*‐g (three members) (Fig. S2). Chromosomes VI and VII both contain a single full‐length *TPS* gene, *CsTPS23* and *CsTPS24* respectively, and the partial *CsTPS27* is located on chromosome VII. *CsTPS23* and *CsTPS24* are classified as *TPS*‐c and *TPS*‐e/f, respectively, and most likely encode COPALYL DIPHOSPHATE SYNTHASE and KAURENE SYNTHASE. Gene models associated with the MEP and MVA pathways or encoding IPP isomerases and prenyl‐transferases were found to be located across all chromosomes and not specifically in the proximity of *TPSs*.

Based on amino acid sequence similarity to representative TPSs from Arabidopsis and tomato, CsTPS1*‐*11 were tentatively classified as monoterpene synthases, CsTPS12*‐*22 as sesquiterpene synthases and CsTPS23 and CsTPS24 as diterpene synthases. Most TPSs contain elements known to be conserved in TPS, such as RRX8W, RXR, DDXXD and NSE/DTE motifs (Table S4). Plastid transit peptides were predicted for CsTPS1‐3, CsTPS9‐11 and CsTPS24 – supporting their putative role as monoterpene synthases or diterpene synthase (CsTPS24) – while a mitochondrial targeting peptide was predicted for CsTPS4. A secretory pathway signal peptide was predicted for CsTPS5. Remarkably, a plastid transit peptide was predicted for the putative sesquiterpene synthase CsTPS15, while no signal peptides were predicted for putative monoterpene synthases CsTPS6‐8 and diterpene synthase CsTPS23.

### Transcriptional responses of the terpenoid biosynthetic module upon herbivory

The effect of herbivory on the expression of the terpenoid biosynthetic genes was analysed using the spider mite‐induced leaf transcriptome dataset presented by He *et al*. (2020). Transcripts of most of the annotated genes involved in the MEP and MVA pathways were present in leaves of genotypes differing in TSSM susceptibility (Fig. 2). Multiple genes in both pathways producing the precursors for terpene biosynthesis were regulated during early TSSM infestation. *3‐HYDROXY 3‐METHYLGLUTARYL‐CoA REDUCTASE* (*HMGR*) in the MVA pathway and *1‐DEOXY‐D‐XYLULOSE‐5‐PHOSPHATE SYNTHASE* (*DXS*) in the MEP pathway were strongly induced in genotype ‘Chinese Long’, which was least susceptible to TSSM, while they were repressed in the susceptible genotype ‘Corona’ (Figs 2, S3). A similar transcriptional response was found for two of the four *GERANYLGERANYL DIPHOSPHATE SYNTHASEs* (*GGPPSs*) while *GERANYL DIPHOSPHATE SYNTHASE* (*GPPS*) and *FARNESYL DIPHOSPHATE SYNTASE* (*FPPS*) were regulated similarly in both genotypes.

**Fig. 3:**
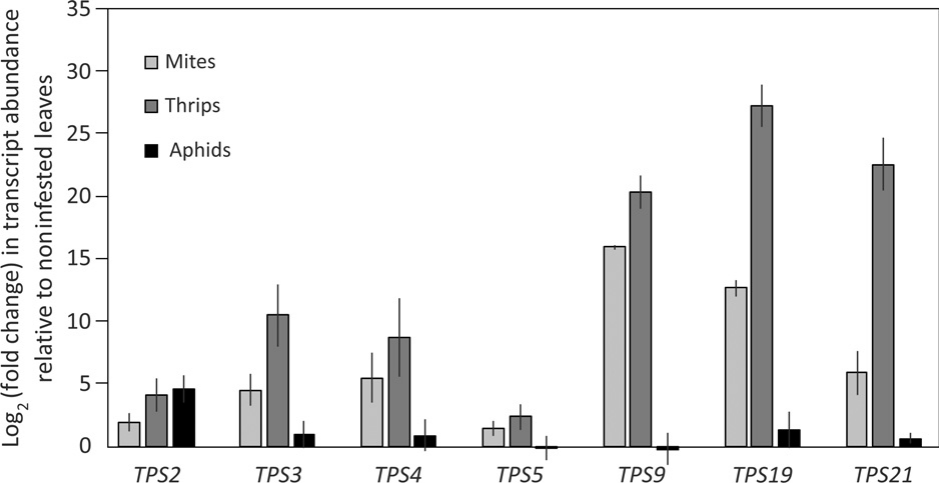
Induction of terpene synthases (TPS) expression in *Cucumis sativus* upon herbivory. Quantitative reverse transcription polymerase chain reaction analysis of the expression of selected *TPS* genes in cucumber leaves (genotype ‘Corona’) that were infested with spider mites (light grey bars), thrips (dark grey bars) or aphids (black bars) for 3 d. Bars represent the expression relative to that in control leaves. Expression was normalized to the expression of reference gene *CsACTIN*. Data are means ± SD of five independent biological replicates.

In control leaves, *TPS5* showed the highest expression in both genotypes, but overall expression of the *TPS* genes was low, at 2.5–5.8% of the average overall gene expression (Table S5). TSSM feeding increased the expression of several *TPSs* (Fig. 2). Quantitative PCR analysis confirmed that TSSM induced *CsTPS9* and *CsTPS19*, and to a lesser extent *CsTPS2‐5* and *CsTPS21* (Figs 3, S3). Thrips feeding induced higher *TPS* expression than TSSM, with *CsTPS9*, *CsTPS19* and *CsTPS21* as the most strongly induced genes. By contrast, aphid feeding resulted only in some induction of *CsTPS2* expression and, to a lesser extent, of *CsTPS3‐4*, *CsTPS19* and *CsTPS21*.

### Characterization of TPS

**Fig. 4:**
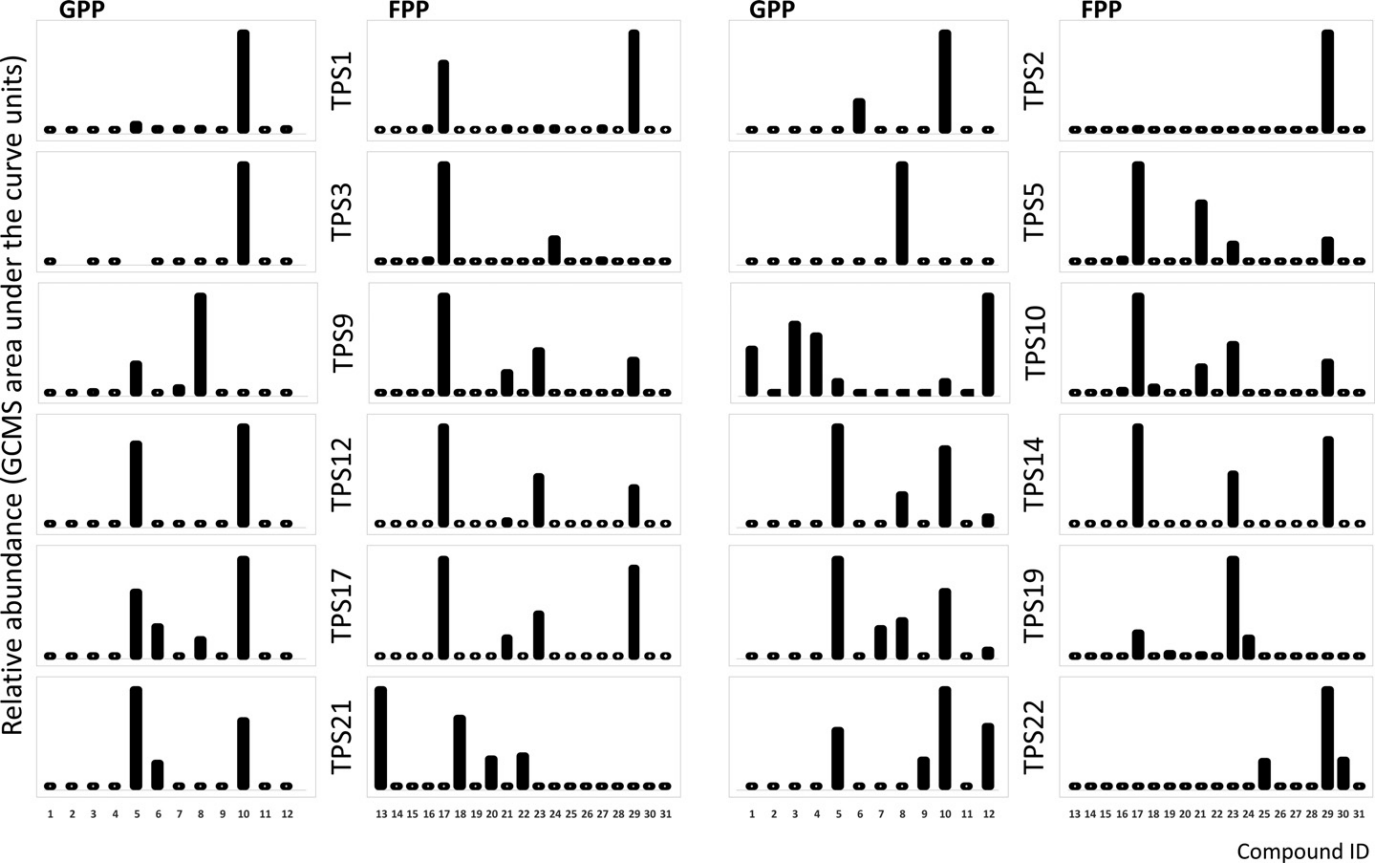
Products formed *in vitro* by CsTPS2, CsTPS3, CsTPS5, CsTPS9, CsTPS10, CsTPS19, CsTPS21 and CsTPS22 upon incubation with geranyl diphosphate (GPP) or farnesyl diphosphate (FPP). Bars indicate the relative abundance of reaction products. Compounds are indicated in numbers are as follows: 1, α‐pinene; 2, α‐phellandrene; 3, sabinene; 4, β‐pinene; 5, myrcene; 6, limonene; 7, (*Z*)‐β‐ocimene; 8, (*E*)‐β‐ocimene; 9, terpinolene; 10, linalool; 11, unknown monoterpene (calculated retention index 1169); 12, α‐terpineol; 13, (*E*)‐caryophyllene; 14, (*Z*)‐β‐farnesene; 15, α‐bergamotene; 16, geranyl acetone; 17, (*E*)‐β‐farnesene; 18, α‐humulene; 19, unknown sesquiterpene (calculated retention index 1485); 20, unknown sesquiterpene (calculated retention index 1490); 21, (*Z;E*)‐α‐farnesene; 22, unknown sesquiterpene (calculated retention index 1500); 23, (*E;E*)‐α‐farnesene; 24, β‐bisabolene; 25, cadinene; 26, (*Z*)‐nerolidol; 27, (*Z*)‐α‐bisabolene; 28, unknown sesquiterpene (calculated retention index 1556); 29, (*E*)‐nerolidol; 30, cadinol; 31, epi‐α‐bisabolol. Supporting Information Fig. S4 shows the GC‐MS chromatograms for CsTPS2, CsTPS9 and CsTPS19 incubated with different substrates. Product profiles of other enzymes that were characterized can be found in Table S6.

Nineteen TPS, including all TSSM‐induced *TPSs*, except for *CsTPS4*, were successfully cloned and heterologously expressed in *E. coli* (Fig. 4; Table S6). All heterologous TPSs, except CsTPS13 and CsTPS18, accepted substrates GPP and FPP, resulting in the formation of various mono‐ and sesquiterpenes, respectively. CsTPS proteins with a predicted chloroplast‐target peptide efficiently produced one or multiple monoterpenoids. These enzymes also catalysed the formation of minor amounts of sesquiterpenes from FPP.

CsTPS1‐3 have predicted chloroplast‐target peptides and produced predominantly linalool when GPP was supplied as substrate. Furthermore, CsTPS9 catalysed the formation of (*E*)‐β‐ocimene and myrcene, and small amounts of sabinene and (*Z*)‐β‐ocimene from GPP. CsTPS11 and CsTPS15 both catalysed the formation of myrcene, limonene, (*E*)‐β‐ocimene, linalool and α‐terpineol from GPP in different amounts and/or ratios, and CsTPS11, in addition, catalysed the formation of an unidentified monoterpene (LRI 1169). The product profile of CsTPS10 was distinct from the other chloroplast‐targeted enzymes as it produced α‐pinene, α‐phellandrene, sabinene, β‐pinene, myrcene, linalool and α‐terpineol from GPP. Despite the presence of a predicted chloroplast‐target peptide, CsTPS1‐3, CsTPS9‐11 and CsTPS15 catalysed the formation of minor amounts of sesquiterpenes from FPP.

We were not able to clone CsTPS6‐8, CsTPS16 and CsTPS20, all genes without a targeting sequence. CsTPS13 and CsTPS18 were not active in any of the assays that we performed. Other proteins without a predicted targeting peptide were CsTPS12, CsTPS14 and CsTPS17, which all catalysed the formation of (*E*)‐β‐farnesene, and to a lesser extent (*E,E*)‐α‐farnesene and (*E*)‐nerolidol from FPP. CsTPS19 predominantly catalysed the formation of (*E,E*)‐α‐farnesene from FPP, consistent with Mercke *et al*. (2004), but produced also traces of (*E*)‐β‐farnesene, (*Z,E*)‐α‐farnesene, bisabolene and an unknown sesquiterpene (LRI 1485). CsTPS21 catalysed the formation of (*E*)‐caryophyllene and α‐humulene from FPP, and the major products of CsTPS22 were (*E*)‐nerolidol and cadinol. When cytosolic TPSs were supplemented with GPP, most of the enzymes produced small amounts of myrcene, limonene and linalool. An exception was CsTPS19 which efficiently catalysed the formation of (*E*)‐β‐ocimene, myrcene and linalool from GPP. CsTPS19 also accepted GGPP to produce the diterpenoid geranyl linalool (Fig. S4), confirming CsTPS19 to be an efficient mono‐, sesqui‐ and diterpene synthase. Both CsTPS23 and CsTPS24 were predicted to encode a diterpene synthase, but only CsTPS24 accepted GGPP to produce geranyl linalool. Both enzymes accepted GPP as a substrate to produce (*E*)‐β‐ocimene, linalool and myrcene in minor amounts. CsTP24 produced a small amount of cadinol and both enzymes produced (*E*)‐nerolidol from FPP.

### Induction of herbivore‐inducible TPS results in circadian enzymatic activity

**Fig. 5:**
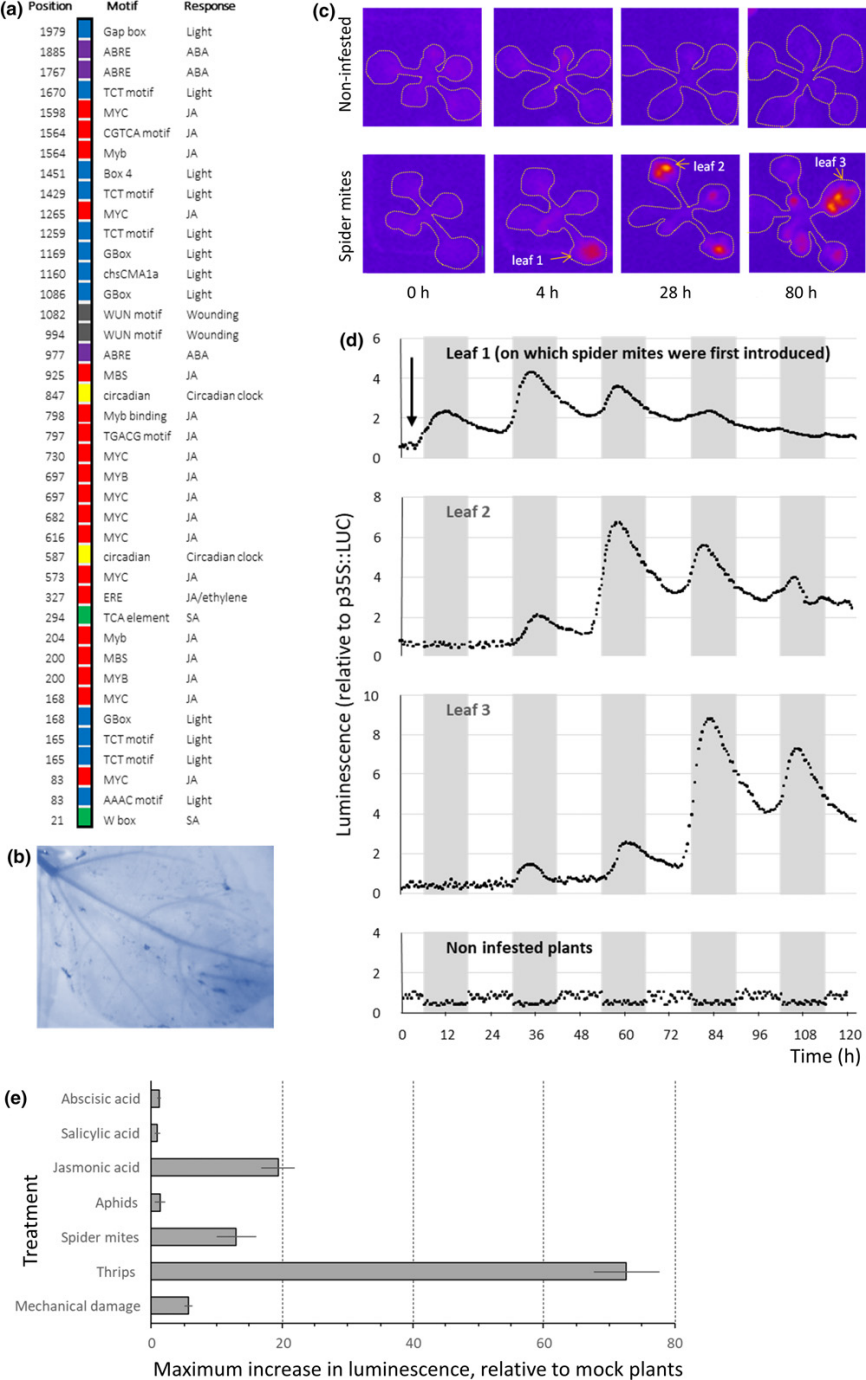
Activity of p*CsTPS9*::ffLUC Arabidopsis reporter plants. (a) Putative *cis*‐acting regulatory elements identified in the upstream 2 kb sequence of CsTPS9. (b) Trypan blue staining indicates successful leaf infestation, 2 d after the introduction of spider mites. (c) Firefly‐LUCIFERASE profile of 4‐wk‐old p*CsTPS9*::LUC reporter plants that were infested with spider mites or were left uninfested. Selected pictures that were taken 4, 28 and 80 h after the introduction of two adult spider mites on leaf 1. (d) Diurnal firefly‐LUCIFERASE (ffLUC) activity profile of leaf 1, leaf 2 and leaf 3 as indicated in panel (c) during five subsequent days under 12 h : 12 h, light : dark cycles. Data are relative luminescence of individual leaves, measured every 20 min; luminescence of p*CsTPS9*/p*35S* was set to 0 at the start of the experiment; E, maximum increase in ffLUC activity of reporter plants (relative to similarly treated p*35S*::ffLUC plants) upon treatment. Maximum ffLUC activity was observed after 24 h for abscisic acid, 24 h for salicylic acid, 24 h for jasmonic acid, 5 h after introduction of a single adult female thrips, 75 h after introduction of two adult female spider mites, 96 h after introduction of two wingless adult aphids and 4 h after repetitive puncturing for 5 min using a needle to inflict mechanical damage. Data are means ± SD of five biological replicates.

*CsTPS2*, *CsTPS9* and *CsTPS19* were selected for further analysis of the regulation of terpene biosynthesis upon herbivory. Multiple *cis*‐acting regulatory elements (CAREs) located in the 2000 bp sequences upstream of the initiation start of these genes, considered to represent the promoter (p*CsTPS*), were identified as responsive to stress‐related phytohormones JA, SA and ABA (Fig. 5a; Table S7). The number of these motifs in p*CsTPS19* was about half of those of p*CsTPS2* and p*CsTPS9* (Table 1). Furthermore, the promoters contained multiple motifs related to light responsiveness and circadian rhythmicity (Table 1; Fig. 5a), suggesting that these p*CsTPSs* may be regulated by photoperiod in addition to JA, SA and ABA.

**Table 1:** Percentage of *cis*‐acting element motifs in the 2000 bp sequence upstream of the translational start of *Cucumis sativus CsTPS2*, *CsTPS9* and *CsTPS19* annotated to be involved in the indicated responsiveness.

	Percentage of motifs in promoter sequence		
Keyword	*CsTPS2*	*CsTPS9*	*CsTPS19*
Light	39.5	30.0	45.8
Circadian	2.3	5.0	4.2
Wounding	–	5.0	4.2
Jasmonic acid	46.5	47.5	33.3
Salicylic acid	7.0	5.0	8.3
Abscisic acid	4.7	7.5	4.2
Total	100	100	100

The expression of *CsTPS2*, *CsTPS9* and *CsTPS19* was very low in nonchallenged cucumber leaves (Table S5), and also in nonchallenged roots and flowers (Li *et al*., 2011). Indeed, nonchallenged transgenic Arabidopsis reporter plants harbouring *CsTPS* promoter regions driving a dual *β*‐*GLUCURONIDASE* (GUS) and *ffLUC* (p*CsTPS::GUS/ffLUC*) showed no blue colour upon histochemical β‐glucuronidase (GUS) staining in roots, leaves, flowers or siliques (Fig. S5).

The herbivore species used in our study all accept Arabidopsis as host (Zhurov *et al*., 2014; Kloth *et al*., 2015; Thoen *et al*., 2016). Visual damage caused by TSSM feeding could be observed after 2 d as white spots, mostly near the veins, and the occurrence of dead cells was confirmed by trypan blue staining (Fig. 5b). Thrips feeding resulted in silver damage as a result of collapsed cells, first visible at 2 d after the onset of feeding. Aphid infestation did not inflict visual damage but infestation was considered to be successful as offspring were present at 3 d after introduction of the aphids.

β‐Glucuronidase staining of p*CsTPS9::GUS/ffLUC* reporter plants showed that expression of the reporter gene was absent in noninfested plants, except for the cotyledons, which in some plants stained blue (Fig. S5). Upon TSSM feeding, leaves stained blue in a patchy pattern corresponding to the damage spots inflicted by the mites. Stained cells in these infested areas were mostly located in the mesophyll layer. Some of the younger leaves that were not damaged by TSSM showed minor staining in the petioles and the veins. By contrast, TSSM‐infested p*CsTPS19* reporter plants showed only blue colouring in local infested leaves, and no blue colouring was observed in TSSM‐infested p*CsTPS2* reporter plants. Thrips infestation resulted in a stronger response of p*CsTPS9* and p*CsTPS19* reporter plants compared with TSSM, but, similar to TSSM feeding, p*CsTPS9* reporter plants showed systemic induction of reporter activity while that of p*CsTPS19* plants was mostly local. Minor blue colouring was observed in the small veins of p*CsTPS2* plants after 96 h of aphid infestation, but not in aphid‐infested p*CsTPS9* and p*CsTPS19* reporter plants.

To better visualize the dynamics of promoter activation, we used luminescence monitoring. Luminescence increased in p*CsTPS9* reporter plants within 1 h after the introduction of a single thrips, and a 73‐fold increase was observed at the end of the second light period (Fig. 5e). After recording luminescence, we observed that the originally infested leaf was seriously damaged by thrips and a number of other leaves showed silver damage spots as well. By contrast, aphids did not cause any detectable induction of luminescence in the p*CsTPS9* reporter plants (Fig. 5e). Visual observation showed that aphids walked around for *c.* 2 h and then remained in the same position, implying they were probing/feeding (Kloth *et al*., 2015). After 96 h no damage was visible on the aphid‐infested plants.

**Fig. 6:**
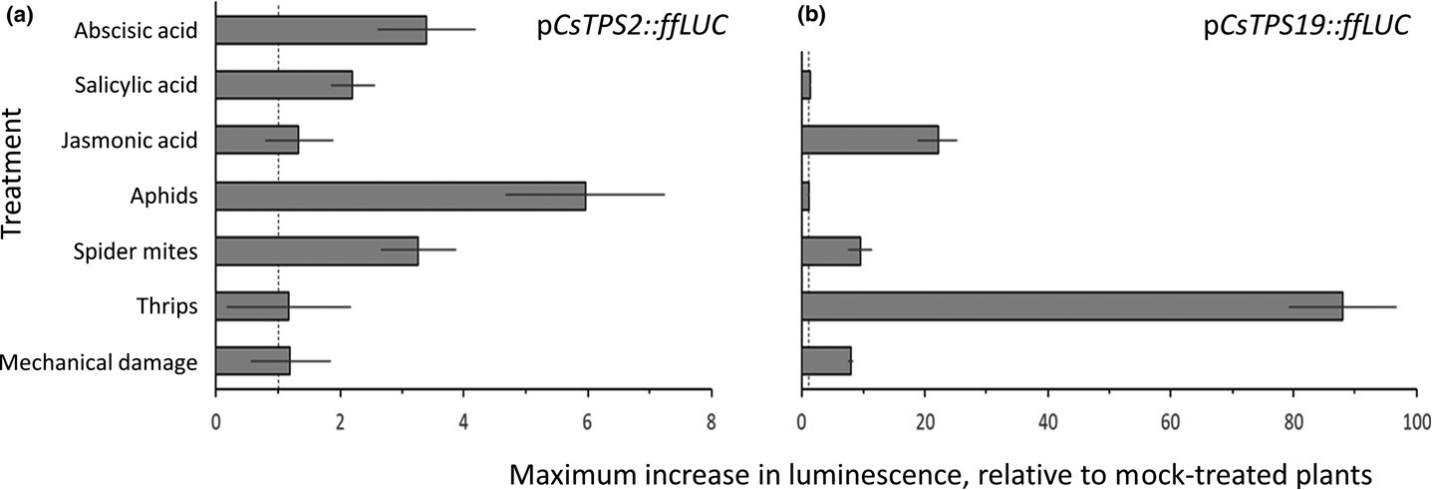
Activity of p*CsTPS2*::ffLUC (a) and p*CsTPS19*::ffLUC (b) Arabidopsis reporter plants. Maximum increase in ffLUC activity of reporter plants (relative to mock‐treated p*35S*::ffLUC plants). Time indicates the period after treatment until maximum activity was observed: abscisic acid (24 h), salicylic acid (24 h), jasmonic acid (24 h), single adult female thrips (5 h), two adult female spider mites (73 h), two wingless adult aphids (96 h), mechanical damage inflicted by repetitive puncturing for 5 min using a needle (4 h). Data are means ± SD of five biological replicates. Note the different scaling of the *x*‐axis for both reporters. The dashed line indicates no increase (i.e. ‘1’).

Luminescence in p*CsTPS19* reporter plants increased upon TSSM and thrips feeding but not aphid feeding (Fig. 6), similar to *pCsTPS9*. Also, the greater damage inflicted by thrips infestation resulted in stronger luminescence than as a result of TSSM feeding. Damage‐induced luminescence was only visible locally, at positions where thrips and TSSM had been feeding in p*CsTPS19* reporter plants, while in p*CsTPS9* some systemic luminescence was observed. By contrast, p*CsTPS2* reporter plants only displayed a minor increase in luminescence upon thrips infestation, mechanical damage or JA treatment but were responsive to TSSM and aphid feeding and SA and ABA treatment (Fig. 6a).

## Discussion

### The cucumber *TPS* gene family is relatively small

The *TPSs* constitute a mid‐sized gene family in plants (Chen *et al*., 2011). The *CsTPS* gene family consists of 27 gene models of which 19 encode complete TPS proteins, confirming the study of Wei *et al*. (2016), and hence form a relatively small TPS family compared with other flowering plant species such as Arabidopsis (40 putative *TPS* gene models; 32 putatively full length; Aubourg *et al*., 2002), tomato (44; 29; Falara *et al*., 2011), rice (57; 34; Chen *et al*., 2011) and grape (152; 69; Martin *et al*., 2010). Remarkably, the *TPS* family in apple consists of 55 gene models of which only 10 are functional (Nieuwenhuizen *et al*., 2013). The majority of the cucumber *TPS* genes are organized into four clusters located on three chromosomes, consistent with the clustering of *TPS* genes in other plant species, including Arabidopsis (Aubourg *et al*., 2002), tomato, (Falara *et al*., 2011) and grape (Martin *et al*., 2010). Clustering of metabolism‐associated genes is relatively common, possibly ensuring co‐inheritance to keep biosynthetic pathways complete (Nutzmann & Osbourn, 2014). Furthermore, clustered genes could share similar regulation mechanisms such as through chromatin modification (Wegel *et al*., 2009). *TPSs* were reported to frequently colocalize with *P450* genes (Boutanaev *et al*., 2015). Remarkably, in cucumber only a single *P450* gene and no members of other classes of genes such as glycosyl transferases were found located within or near any of the *TPS* clusters. Just as reported for other species, *CsTPSs* located within the same cluster in the genome were assigned to similar clades in the phylogenetic tree and are hence more homologous to each other, probably as a consequence of tandem duplication. Evolutionary analysis of terpenoid biosynthesis‐related genes and supergene clusters of 17 genomes demonstrated that genes encoding TPSs are more enriched for tandem duplications than genes encoding enzymes involved in the upstream MVA pathway and IPP isomerases (Hofberger *et al*., 2015).

Like many TPSs characterized in other plant species, CsTPSs can accept different substrates. Most CsTPSs that were tested *in vitro* catalysed the formation of multiple terpenes from the same precursor, a common phenomenon in plant TPSs. For example, 10 different monoterpenes were formed by a single Arabidopsis TPS (Chen *et al*., 2004). Most of the characterized tomato TPSs catalysed the formation of more than one terpene (Falara *et al*., 2011). At the same time, some of the terpenes we detected were synthesized by multiple CsTPSs. For example, linalool was the major product of TPS‐g clade CsTPS1, CsTPS2 and CsTPS3, and also a minor product of most of the other CsTPSs. Linalool is a common floral and foliar volatile with two distinct enantiomers that have distinct roles in pollinator attraction and plant defence (Raguso, 2016; He *et al*., 2019). Linalool enantiospecific enzymes have been identified in, for example, Arabidopsis, producing (R)‐(−) and (S)‐(+)‐linalool, respectively, as their major products (Ginglinger *et al*., 2013). Whether cucumber leaves and flowers emit specific isomers is unknown and the present study did not allow us to distinguish between both enantiomers. Further studies might also investigate whether CsTPS1‐3 contribute to enantiomeric‐specific linalool formation, if any, and its specific role in plant–arthropod interactions.

Most cucumber TPSs convert GPP and FPP to acyclic mono‐ and sesquiterpenes while the formation of cyclic terpenes was catalysed by a limited number of CsTPSs only, including CsTPS10 which catalyses the formation of a pinyl cation en route to the formation of β‐pinene, α‐pinene, sabinene and α‐phellandrene. The root‐specific CsTPS11 was also demonstrated to use a pinyl cation as intermediate in the formation of cyclic terpenes (Wei *et al*., 2016). Other cyclic sesquiterpenes, including (*E*)‐caryophyllene, α‐humulene and cadinol, were produced by just a few CsTPSs.

Depending on the presence of terpenoid precursors in different cell compartments, the product profile of CsTPSs *in planta* may differ from those *in vitro*. Previously, we demonstrated that targeting a nerolidol synthase from strawberry to different cell compartments in Arabidopsis determined the abundance and ratio of mono‐ and sesquiterpenoid products, confirming the importance of precursor availability for product formation (Aharoni *et al*., 2004; Kappers *et al*., 2005; Houshyani *et al*., 2013). Accordingly, the predicted subcellular localization of cucumber TPSs coincides with the presence of the precursors which they effectively use. An exception is CsTPS19, which accepts GPP, FPP and GGPP efficiently to produce (*E*)‐β‐ocimene, (*E,E*)‐α‐farnesene and geranyl linalool, respectively, supporting its role as a genuine multiple‐function TPS. Thus, the enzymatic activity of the CsTPSs in combination with their subcellular localization and their expression together determine which terpene compounds are produced in cucumber under which conditions.

### Potential roles of CsTPSs in herbivore‐induced volatile formation

The volatile blend of control cucumber leaves contained few terpenoids in low amounts, including limonene, (*E*)‐β‐ocimene and linalool, coinciding with expression of *CsTPS1‐3* and *CsTPS5* in these leaves. Upon TSSM and thrips feeding, the expression of *CsTPS2‐5*, *CsTPS9*, *CsTPS19* and *CsTPS21* increased, suggesting a role for these genes in the biosynthesis of volatile terpenoids induced by cell‐content feeders, which are mainly associated with JA‐related signalling (Zhurov *et al*., 2014; Steenbergen *et al*., 2018). Previous studies documented the volatile blends emitted by different cucumber genotypes upon TSSM feeding (Takabayashi *et al*., 1994; Bouwmeester *et al*., 1999; Agrawal *et al*., 2002; Mercke *et al*., 2004; Kappers *et al*., 2010, 2011; He *et al*., 2020). The main components of these induced blends are terpenoids, including (*E*)‐β‐ocimene, linalool, DMNT, (*E,E*)‐α‐farnesene and TMTT, common constituents of many floral and herbivore‐induced plant volatile bouquets with various functions in different plant–arthropod interactions, depending on the context (Dicke *et al*., 1990, 1990,1990, 1990; Tholl, 2006; He *et al*., 2019; Burdon *et al*., 2020).

Although thrips infestation resulted in more damage and a higher total volatile emission compared with that of TSSM, the composition of the terpenoid blend was comparable. By contrast, upon feeding by aphids, which mainly induces SA signalling (Moran & Thompson, 2001), the terpenoid blend differed both quantitatively and qualitatively from that of TSSM‐ and thrips‐damaged plants.

Considering the multiple minor products that are produced by CsTPSs besides their major products, induction of these genes enables the plants to produce a wide spectrum of volatiles and fine‐tune their volatile signature in response to herbivory. Most of the terpenoids emitted by noninfested and infested leaves correlated well with the product profiles of the CsTPSs and the expression of the corresponding genes. An exception was the increased emission of α‐pinene, α‐phellandrene and sabinene by leaves infested with cell‐content feeding herbivores, while the gene that encodes the most likely corresponding terpene synthase (CsTPS10) was not upregulated. Genes associated with the biosynthesis of terpenoid precursors upstream of the TPSs were also found to be differentially regulated, and this might have implications for the availability of precursors for constitutively expressed *TPSs* in different cell organelles. Hence, the final terpenoid metabolite profile will be determined by *TPSs* that are induced upon herbivory as well as those that are constitutively expressed. Cucumber genotypes previously characterized for their herbivore‐induced plant volatiles emit mostly similar compounds with different abundances that consequently affected the level of indirect defence (Kappers *et al*., 2010, 2011).

Further fine‐tuning of the volatile signature in response to different herbivore feeding will have consequences for multitrophic interactions. Although we did not compare the different herbivore‐induced volatile blends regarding the attractiveness of these odours towards natural enemies, natural enemies can distinguish different blends of terpenoid volatiles upon infestation by different herbivorous arthropods. For instance, lima bean plants emitted different volatile blends as a result of feeding by *Spodoptera exigua* and *T. urticae* and, consequently, *P. persimilis* predators were more attracted to plants infested by their prey, *T. urticae* (de Boer *et al*., 2004). When lima bean and cucumber plants were infested by both herbivores separately or together, the plants emitted different amounts of volatile compounds, including several terpenes, and the dual‐infested plants were more attractive to predatory mites than those damaged by only a single herbivore species (De Boer *et al*., 2008).

### Involvement of stress‐related phytohormones in the response of CsTPS to herbivores with different feeding guilds

Two‐spotted spider mites and thrips activated transgenic Arabidopsis p*CsTPS9* and p*CsTPS19* reporter plants, while aphids and TSSM activated p*CsTPS2* reporter plants, indicating that the promoters of *CsTPS9* and *CsTPS19* respond to cell‐content feeders. As reporter activity of these plants was also activated by mechanical damage, a shared upregulation in the response to TSSM and thrips could be the result of the fact that both herbivores cause mechanical wounding. Indeed, thrips inflicted more damage than TSSM and, correspondingly, thrips induced stronger luminescence in reporter plants. Limited, one‐time, mechanical damage quickly activated the promoter, which then decreased to the control level within 1 d. Repetitive mechanical damage of lima bean plants using an artificial caterpillar resulted in an induced volatile blend that was strikingly similar in quality to the blend induced by herbivore feeding (Mithofer *et al*., 2005), suggesting that repetitive mechanical damage inflicted by herbivory is sufficient to trigger the biosynthesis of herbivore‐inducible volatiles in plants. Phloem‐feeding aphids inflict less damage, as they navigate their stylets between the cell walls to reach phloem vessels with limited harming of cell integrity (Tjallingii & Hogen Esch, 1993; Kloth *et al*., 2016). Comparison of the up‐ and downregulated genes in Arabidopsis infested by herbivores with different feeding habits showed that similar transcriptional responses were induced by chewing generalist species *Plutella xylostella* and *Spodoptera litoralis*, while generalist *F. occidentalis* and phloem‐feeding generalists *Bemisia tabaci* and *M. persicae* caused more and different transcriptional changes compared with *P. xylostella* (Reymond *et al*., 2004; de Vos *et al*., 2005; Kempema *et al*., 2007; Kusnierczyk *et al*., 2007; Little *et al*., 2007; Ehlting *et al*., 2008).

Both p*CsTPS9* and p*CsTPS19* reporter plants were responsive to JA but not to SA and ABA. The JA/ethylene pathway is activated in response to thrips feeding (Steenbergen *et al*., 2018) and TSSM infestation in multiple species, including lima bean (Dicke *et al*., 1999), tomato (Ament *et al*., 2004) and cotton (Miyazaki *et al*., 2014), although a recent study showed that, unlike adults, juvenile TSSM induce SA but not JA defences in tomato (Liu *et al*., 2020). Endogenous JA and SA increased within hours after the onset of TSSM infestation in Arabidopsis (Zhurov *et al*., 2014) and *Capsicum* (Zhang *et al*., 2020). In cucumber, JA induces a blend of volatiles that is qualitatively similar to the blend induced by TSSM (Kappers *et al*., 2010). Furthermore, methyl‐SA was emitted upon TSSM herbivory by multiple plant species, including lima bean (Dicke *et al*., 1990, 1990,1990, 1990), tomato (Ament *et al*., 2004) and cucumber (Kappers *et al*., 2011). Neither SA nor ABA application triggered any response of the reporters driven by p*CsTPS9* or p*CsTPS19*, and although the SA‐regulation network may play a role, it appears that JA dominates the regulation of these TPSs that are part of the inducible defence to cell‐content feeders.

Interestingly, the promoter activity of p*CsTPS2* reporter plants was triggered by aphids and TSSM, and by SA and ABA application, whereas JA only provoked minimal promoter activity in these plants. Aphids are known to induce formation of ABA, and ABA‐regulated genes are over‐represented among genes that are induced by *M. persicae* saliva infiltration into Arabidopsis leaves (Hillwig *et al*., 2016). Feeding by the carmine spider mite *T. cinnabarinus* altered ABA content in tomato plants (Gawrońska & Kiełkiewicz, 1999). Furthermore, SA‐regulated transcripts increase upon aphid feeding (Moran & Thompson, 2001), although this response is very local (de Vos *et al*., 2005). The induction of the *CsTPS2* promoter by TSSM and aphids could be explained via the presence of ABA‐ and SA‐responsive elements in this promoter, suggesting that ABA and SA are important for regulation of *CsTPS2* and its contribution to the aphid feeding‐induced volatile blend. Multiple CAREs present in the promoter sequences of *CsTPS2*, *CsTPS9* and *CsTPS19* were identified as possibly involved in JA, SA or ABA responsiveness. For example, G‐boxes (CACGTG), required for JA‐mediated expression regulation (Kim *et al*., 1992; Endt *et al*., 2007), W‐boxes (TTGACC) associated with responsiveness to SA (Li *et al*., 2006) and ABRE motifs, related to ABA responsiveness (Lenka *et al*., 2009), were present in all three promoters. The wound‐responsive WUN motif was found in p*CsTPS9* and p*CsTPS19* but not in p*CsTPS2*, and this might play an as‐yet‐unknown role in the different responses to the mechanical damage inflicted by herbivores from different feeding guilds. Whether motifs in these promoters really function as binding sites to potential transcription factors, and which conditions render specific CAREs indispensable for promoter activity are still unclear and were not the purpose of our study. However, the presence of these motifs probably allows promoters to be bound by transcription factors induced through JA, SA or ABA signalling, hence regulating the volatile blend resulting from hormonal crosstalk.

### Regulation of CsTPS by light and the circadian clock

The observed rhythmic oscillation in luminescence might be the result of herbivore behaviour, as they often display rhythmic feeding. For example, *T*. *ni* caterpillars show diurnal feeding behaviour (Goodspeed *et al*., 2012). However, regardless of whether feeding behaviour of the herbivores in this study was circadian, the nocturnal maximum activity of reporter plants upon JA treatment demonstrates that the expression of the studied *CsTPSs* displays circadian rhythmicity.

Rhythmic emission of volatiles and expression of genes involved in their biosynthesis have been reported in multiple species. Methyl‐JA‐induced emission of terpenes and methyl‐SA from Norway spruce displayed a diurnal rhythm (Martin *et al*., 2003). Lima bean leaves mechanically damaged during the day emitted maximum amounts of (*E*)‐β‐ocimene and (*Z*)‐3‐hexenyl acetate in the late photo‐phase, while nocturnally applied mechanical damage triggered nocturnal emission of (*Z*)‐3‐hexenyl acetate but only minor amounts of (*E*)‐β‐ocimene, which burst after onset of the photo‐phase (Arimura *et al*., 2008). *Phaseolus vulgaris* plants released trace amounts of volatiles with no obvious rhythm, but upon infestation with *Liriomyza huidobrensis* larvae, plants released higher amounts of volatiles with a clear rhythm which peaked at the end of the day (Sufang *et al*., 2013). The expression of *Artemisia annua QH6*, encoding a pinene synthase is diurnally regulated (Lu *et al*., 2002) and luciferase activity driven by the *QH6* promoter with a mutated G‐box showed a rhythm lacking a peak in the early morning which was present when the intact G‐box was present (Zhou *et al*., 2015). Multiple light‐associated CAREs are present in the promoters we tested, including the light‐responsive element box I (TTTCAAA) (Yamada *et al*., 1994), and a circadian motif (CAANNNNATC, Piechulla *et al*., 1998). The G‐box present in each of the promoter sequences could be essential for light regulation as well (Lopez‐Ochoa *et al*., 2007).

Our results suggest that the promoters tested induce peak gene expression during the night, while the emission of the corresponding terpenes and green leaf volatiles occurs mainly during the light period (I. F. Kappers, unpublished). Possibly, high nocturnal expression of *CsTPS2*, *CsTPS9* and *CsTPS19* results in the accumulation of active enzymes which are ‘ready to go’ when enough substrate becomes available at the onset of the day to fuel production of energy‐costly secondary metabolites only during the photoperiod. This is in agreement with the burst of emission of (*E*)‐β‐ocimene upon the onset of light by lima bean plants which were damaged in the previous dark period (Arimura *et al*., 2008). In lima bean, the expression of *β‐OCIMENE SYNTHASE* is regulated via JA accumulation at wounded sites and the biosynthesis of (*E*)‐β‐ocimene is dependent on CO_2_ fixation by photosynthesis in the chloroplasts (Arimura *et al*., 2008), where the MEP pathway synthesizing the terpenoid precursors GPP and GGPP is also located. The expression of the genes of the MEP pathway is light‐dependent (Hemmerlin *et al*., 2012) and, indeed, expression of almost all MEP‐pathway genes in Arabidopsis seedlings is repressed in darkness (Hsieh & Goodman, 2005). The expression of MEP‐pathway genes encoding 1‐DEOXY‐D‐XYLULOSE‐5‐PHOSPHATE SYNTHASE and two GPP SYNTHASES are upregulated in cucumber leaves upon TSSM infestation (He *et al*., 2020). Hence, it is not unlikely that the supply of precursors determines the diurnal emission of terpene volatiles in cucumber leaves triggered by herbivory. To verify this, the rhythmicity of expression of the genes encoding the precursor supply pathways should be evaluated.

In conclusion, we identified the cucumber *TERPENE SYNTHASE* (*CsTPS*) gene family from the sequenced cucumber genome and characterized their role in the production of volatiles in leaves with and without herbivore feeding. We identified the *CsTPS* genes that contribute to the volatile terpenoid blend of cucumber leaves upon feeding by important cucumber pest species with dissimilar feeding guilds and revealed the involvement of stress‐related phytohormones and circadian rhythmicity in the regulation of the production of this terpenoid blend.

## Author contributions

Conceptual design and funding, HJB, MD, IFK, experimental work, JH, FV, AJ, IFK, manuscript: all authors.

## Supporting information


**Fig. S1** Chromosomal position of CsTPS.
**Fig. S2** Phylogenetic tree of CsTPS.
**Fig. S3** Gene expression of selected genes in the terpenoid module.
**Fig. S4** Product profiles of heterologous CsTPS.
**Fig. S5** Histochemical β‐glucuronidase staining.

**Table S1** Primers used in this study.
**Table S2** GC‐MS analysis of volatile emissions.
**Table S3** Annotation of the terpenoid biosynthetic module genes.
**Table S4** Genomic information of *CsTPS* genes.
**Table S5** RPKM values of genes in the terpenoid biosynthetic module.
**Table S6** Heterologous assays.
**Table S7** CARE motif analysis.
Please note: Wiley Blackwell are not responsible for the content or functionality of any Supporting Information supplied by the authors. Any queries (other than missing material) should be directed to the *New Phytologist* Central Office.

## Data Availability

The data that support the findings of this study are available in the Supporting Information of this article.
