# Supplementary material for: Terpene synthases in cucumber (Cucumis sativus) and their contribution to herbivore‐induced volatile terpenoid emission
Source: New Phytol. 2021 Nov 10;233(2):862–77. doi: 10.1111/nph.17814 (PMC9299122; doi:10.1111/nph.17814)
Supplement: Supplementary file 1 — Fig. S1 Chromosomal position of CsTPS. Fig. S2 Phylogenetic tree of CsTPS. Fig. S3 Gene expression of selected genes in the terpenoid module. Fig. S4 Product profiles of heterologous CsTPS. Fig. S5 Histochemical β‐glucuronidase staining. [file NPH-233-862-s001.pdf]

***New Phytologist* Supporting Information**

Article title: Terpene synthases in cucumber (*Cucumis sativus*) and their contribution to herbivore-induced volatile terpenoid emission

Authors: Jun He, Francel Verstappen, Ao Jiao, Marcel Dicke, Harro J. Bouwmeester, Iris F. Kappers

Article acceptance date: 12 October 2021

The following Supporting Information is available for this article:

Fig. S1 Distribution of Cs*TPS* genes in the cucumber genome

Fig. S2 Phylogenetic tree of CsTPS

Fig. S3 Gene expression of selected genes in the terpenoid module

Fig. S4 Product profiles of heterologous CsTPS

Fig. S5 Histochemical β-glucoronidase staining

Table S1 Primers

Table S2 Volatile emissions

Table S3 Annotation of the Terpenoid Biosynthetic Module genes

Table S4 Genomic information of *CsTPS* genes

Table S5 RPKM values of genes in the Terpenoid Biosynthetic Module

Table S6 Heterologous assays

Table S6 CARE motif analysis

(Tables S1–S6, see separate file)

Fig. S1 Distribution of *CsTPS* genes in the cucumber genome. The location of all *CsTPS* genes is indicated at the relative position in seven artificial chromosomes (white bars); the loci harbouring *CsTPS* genes are enlarged, and gene models are shown in black (*CsTPS* genes) or white (non-*CsTPS* genes), with the point representing the orientation of the genes and the Kb number indicating the position in the artificial chromosomes.

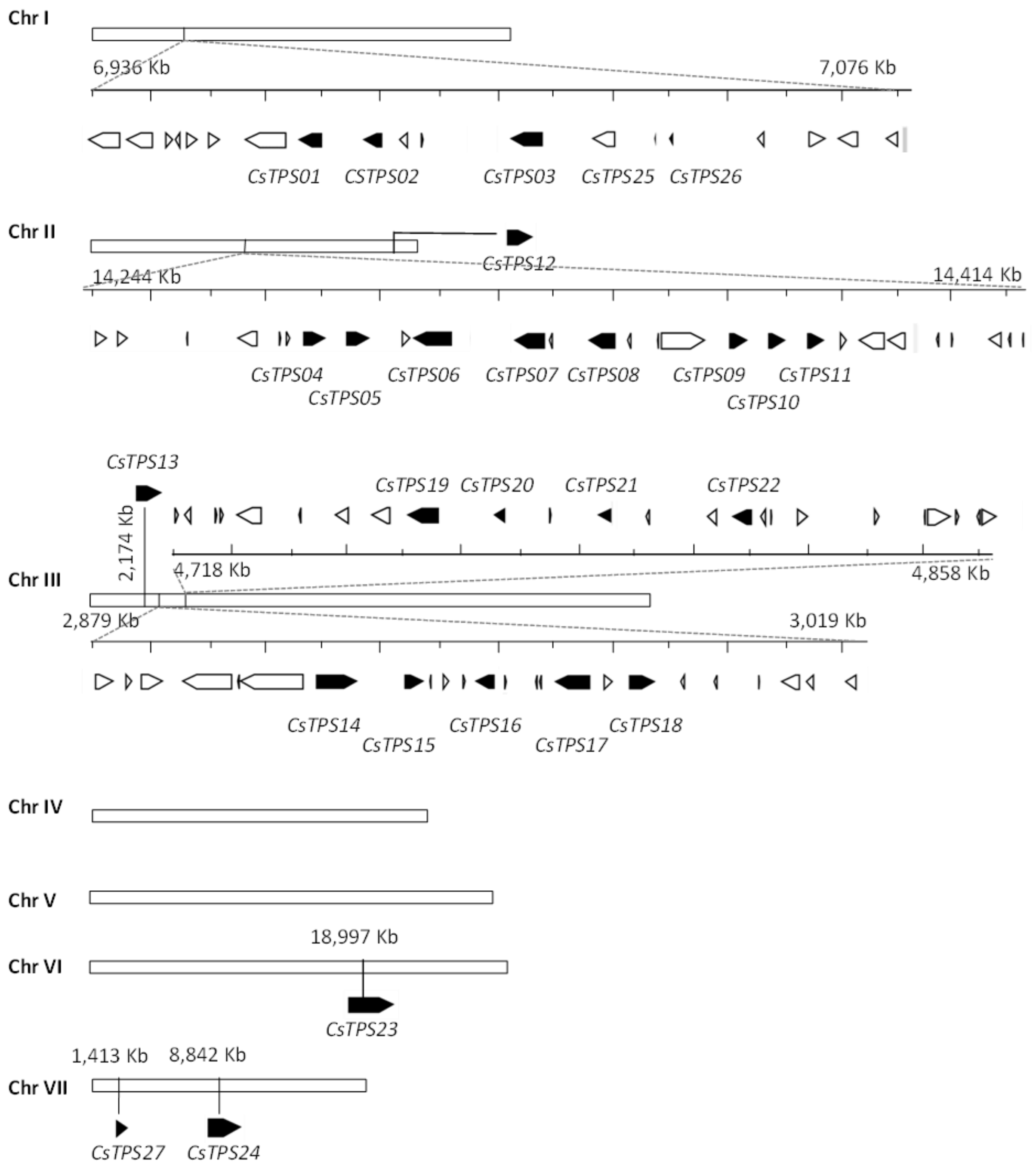

Fig. S2 Phylogenetic relationship of full length CsTPSs from Cucumis sativus. CsTPSs with putative full-length sequences are sub-grouped into TPS-a through TPS-f according to their sequence similarity to reported TPSs in Arabidopsis.

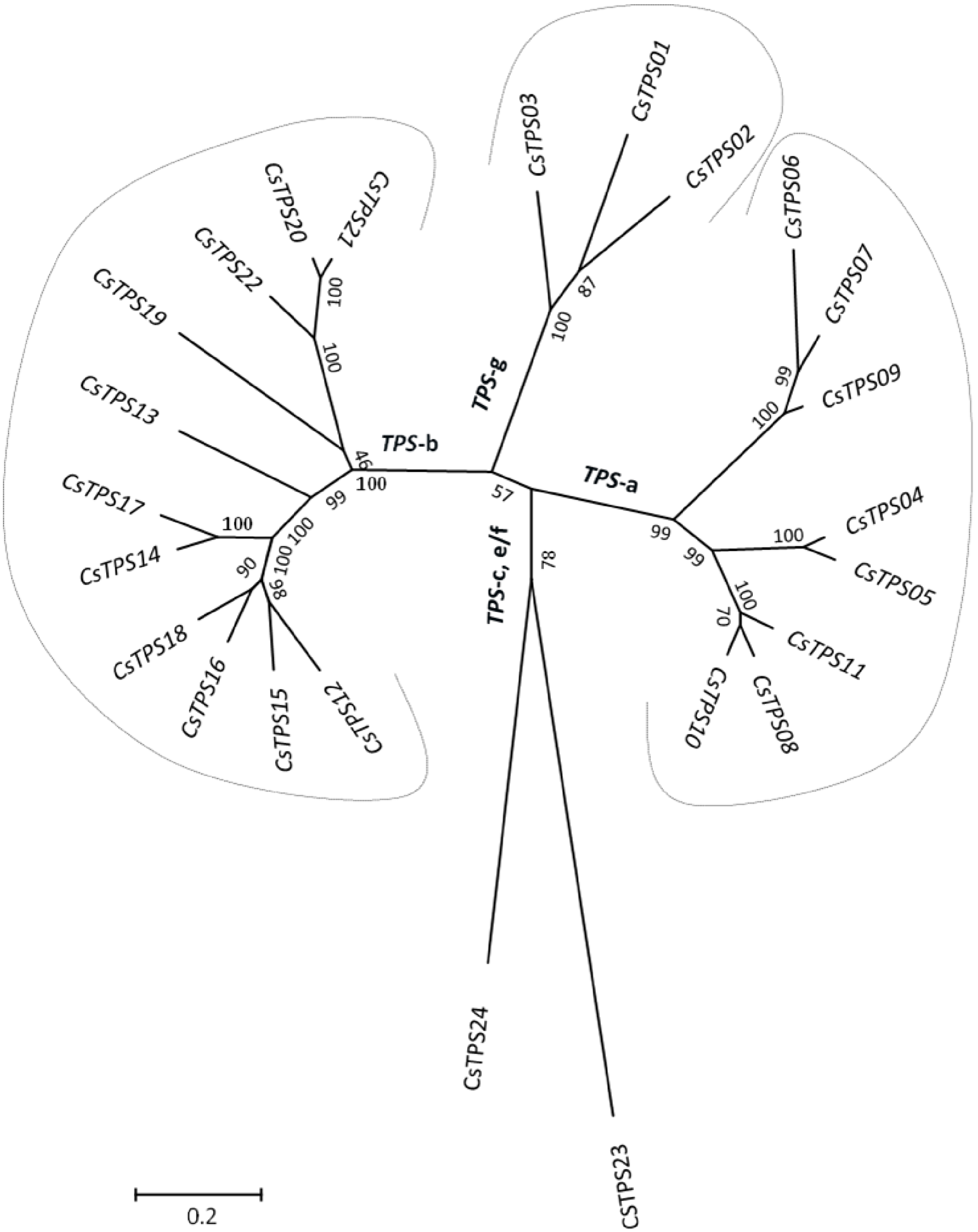

Fig. S3 Quantitative RT-PCR analysis of gene expression in two *Cucumis sativus* genotypes ('Chinese Long, Cl' and 'Corona, Co') upon herbivory. A, relative expression of selected DXS and HMGR genes in cucumber leaves that were infested with spider mites for three days; B, relative expression of *CsTPS2*, *CsTPS9* and *CsTPS19* genes in cucumber leaves that were infested with spider mites (green bars), thrips (blue bars) or aphids (yellow bars) for three days. Bars represent the expression relative to that in control leaves. Expression was normalized to the expression of reference gene *CsACTIN*. Data are means of 5 independent biological replicates ± SD.

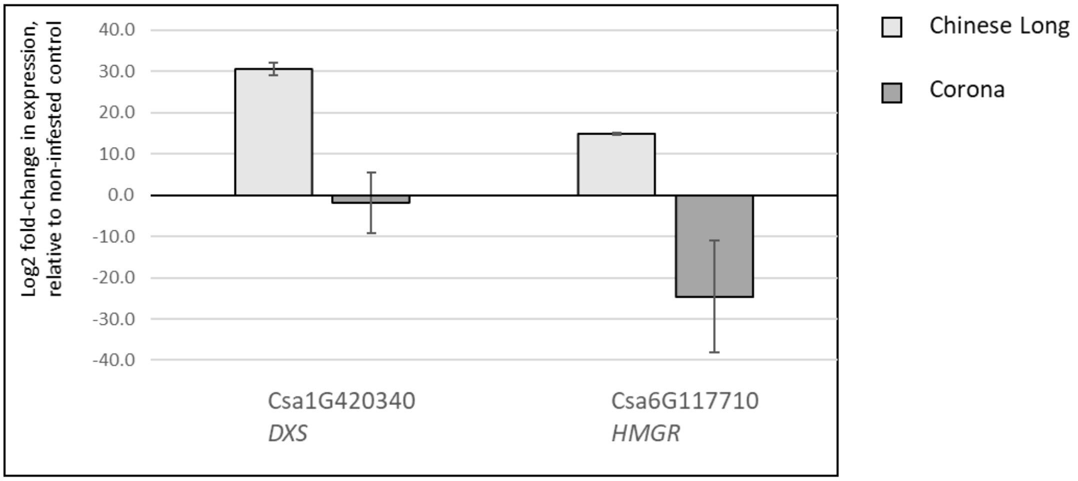

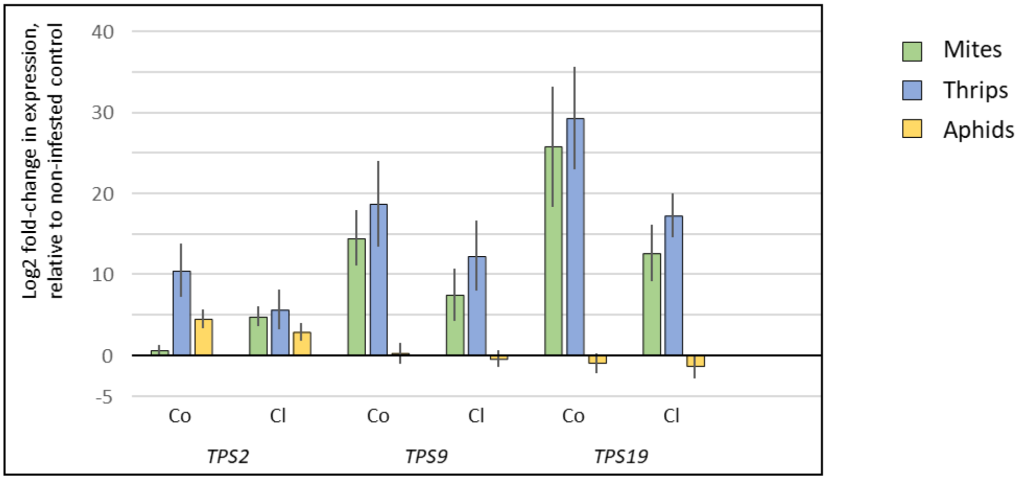

Fig. S4 GCMS chromatograms showing product profiles of heterologous CsTPS2, CsTPS9 & CsTPS19 upon incubation with GPP, NPP, e,e-FPP, z,z-FPP and GGPP

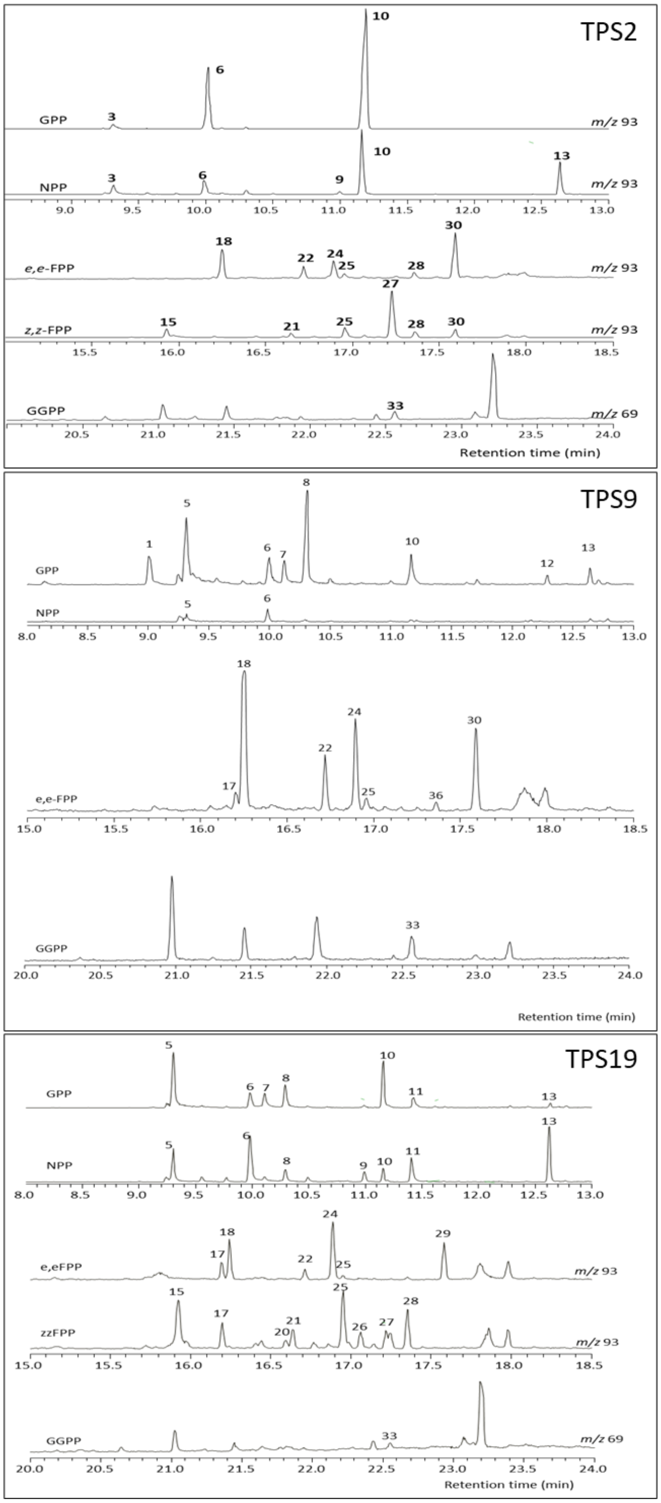

Fig. S5 Arabidopsis reporter plants harbouring GUS::ffLUC driven by the Cucumber *pTPS9* promotor

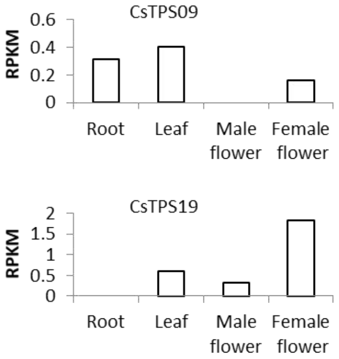

Gene expression in non-stressed cucumber organs Data extracted form Li, Z., Zhang, Z., Yan, P., Huang, S., Fei, Z. and Lin, K. (2011). RNA-Seq improves annotation of protein-coding genes in the cucumber genome. BMC Genomics, 12, 540.

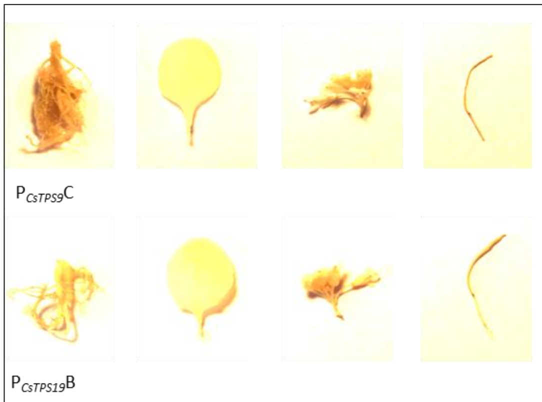

GUS staining of non-stressed transgenic Arabidopsis plants harbouring CsTPS promotors

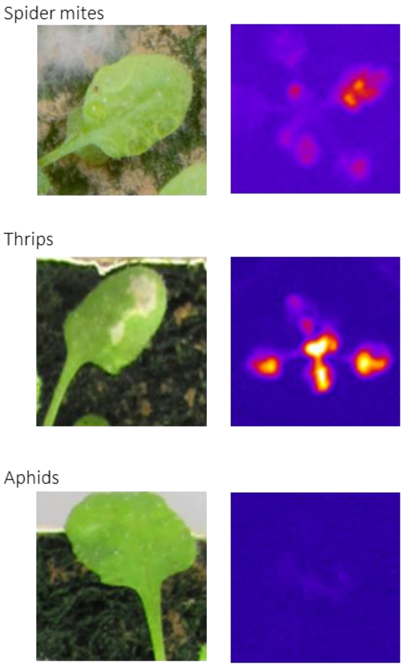

Transgenic Arabidopsis plants harbouring p*CsTPS9::GUS-ffLUC* infested with herbivores with different feeding guild. Left panels show visible damage and right panels show luminescence profile after 3 days of infestation

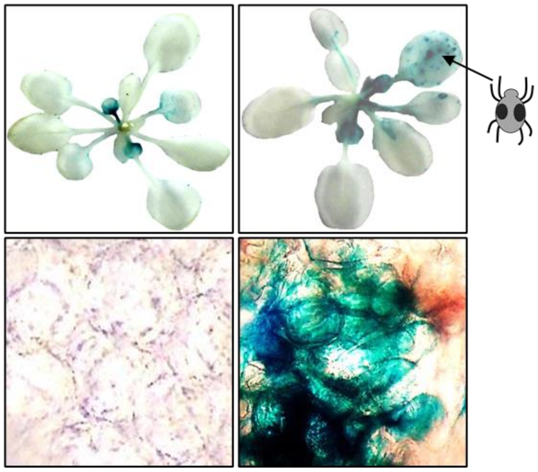

GUS staining of transgenic Arabidopsis plants harbouring pCsTPS9::GUS-ffLUC that were left un-infested (left) or infested with a single spider mite for three days (right). Upper panel shows the whole plant, the lower panel shows mesophyll cells. Arrow indicates where the spider mite was introduced.

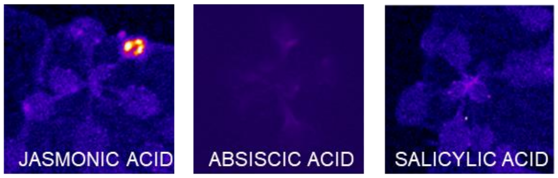

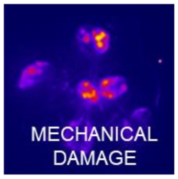

Luminescence of transgenic Arabidopsis plants harbouring p*CsTPS9::GUS-ffLUC* upon different treatments.
